# Massively Parallel Reporter Assays for High-Throughput In Vivo Analysis of Cis-Regulatory Elements

**DOI:** 10.3390/jcdd10040144

**Published:** 2023-03-29

**Authors:** Yanjiang Zheng, Nathan J. VanDusen

**Affiliations:** 1 Key Laboratory of Birth Defects and Related Diseases of Women and Children of MOE, Department of Pediatrics, West China Second University Hospital, Sichuan University, Chengdu 610041, China; 2 Department of Pediatrics, Herman B Wells Center for Pediatric Research, Indiana University School of Medicine, Indianapolis, IN 46202, USA

**Keywords:** MPRA, transcriptional regulation, functional genomics, cardiovascular, development

## Abstract

The rapid improvement of descriptive genomic technologies has fueled a dramatic increase in hypothesized connections between cardiovascular gene expression and phenotypes. However, in vivo testing of these hypotheses has predominantly been relegated to slow, expensive, and linear generation of genetically modified mice. In the study of genomic *cis*-regulatory elements, generation of mice featuring transgenic reporters or *cis*-regulatory element knockout remains the standard approach. While the data obtained is of high quality, the approach is insufficient to keep pace with candidate identification and therefore results in biases introduced during the selection of candidates for validation. However, recent advances across a range of disciplines are converging to enable functional genomic assays that can be conducted in a high-throughput manner. Here, we review one such method, massively parallel reporter assays (MPRAs), in which the activities of thousands of candidate genomic regulatory elements are simultaneously assessed via the next-generation sequencing of a barcoded reporter transcript. We discuss best practices for MPRA design and use, with a focus on practical considerations, and review how this emerging technology has been successfully deployed in vivo. Finally, we discuss how MPRAs are likely to evolve and be used in future cardiovascular research.

## 1. Introduction

The mammalian heart is a complex organ composed of diverse cell types that must undergo specification, differentiation, and maturation [1,2,3]. Collectively, these processes drive morphogenesis and enable mature cardiac function. Abnormalities in heart development can cause congenital heart disease (CHD) [4,5,6] or can contribute to impairments in adult cardiac function, resulting in a diversity of conditions including dilated cardiomyopathy (DCM), hypertrophic cardiomyopathy (HCM), and arrhythmogenic cardiomyopathy (ACM) [7,8,9,10]. The coordination of multiple cardiac lineages during development and adult homeostasis is orchestrated by the precise transcriptional control of gene expression; therefore, developing a firm understanding of how gene expression is controlled and how aberrant gene expression impacts cardiac phenotypes is a crucial first step in the development of targeted therapies. Over the past few decades, studies of cardiovascular gene expression have benefited from a spectrum of approaches for creating genetically modified model organisms, with most mammalian studies being conducted in mice. These murine models have included the generation of transgenics, homologous recombination-mediated systemic gene knockouts, targeted gene knockins, and conditional and inducible gene knockout via the Cre/LoxP system [11,12,13,14]. While these approaches have revealed significant mechanistic insights into the development and function of the heart, they exhibit limitations related to the time and expense required. While the widespread dissemination of CRISPR/Cas gene editing technology within the last decade has expedited the process of generating genetically modified mice [15,16], the one mouse line for one modification paradigm remains too slow and constraining for the efficient systematic characterization of cardiac transcriptional networks. Thus, alternative approaches that prioritize speed, flexibility, and throughput are needed.

Genome-wide association studies (GWAS) indicate that more than 90% of disease-associated genetic variation is located within non-coding regions [17], which include enhancers and promoters. The detection, validation, and functional characterization of disease-associated regulatory elements can expand our understanding of gene regulation and improve our ability to treat human disease. Methodologies such as DNase I hypersensitive site sequencing (DNase-seq) and assay for transposase-accessible chromatin sequencing (ATAC-seq) to identify accessible chromatin regions [18,19], enhancer RNA sequencing [20], and chromatin immunoprecipitation sequencing (ChIP-seq) to reveal DNA occupancy by transcription factors or chromatin markers [21] have all been used to generate numerous *cis*-regulatory element predictions. For instance, large annotation efforts such as the US National Institutes of Health Roadmap Epigenomics Program and ENCODE have uncovered millions of putative regulatory elements within more than 100 human cell types [22,23]. Importantly, all of these methods involve measuring genomic characteristics that correlate with ***cis***-regulatory element activity, but do not directly measure activity. As a result, the vast majority of candidate elements remain functionally uncharacterized. Validation and functional analyses via traditional methods such as transient transgenic reporters, gene-targeted reporters, and enhancer knockout have been used successfully for a subset of elements; however, the low-throughput high-cost nature of these approaches diminishes their utility. Recently, massively parallel reporter assays (MPRAs) have been deployed to bypass these limitations [24,25,26]. MPRAs are a powerful functional genomics technique that utilizes a reporter assay with a sequencing-based readout to measure the activities of thousands of DNA elements in a single experiment.

In this manuscript, we provide an overview of how MPRAs work, and review key points for design, execution, and data analysis. We highlight variations in the MPRA approach, summarizing the strengths and weaknesses of each, and we highlight cardiac-specific considerations. Furthermore, we review the progress that has been made toward adapting MPRAs to in vivo experimentation via viral delivery. Finally, we discuss the limitations of the MPRA technique and how MPRAs are likely to evolve and be used in future cardiovascular research.

## 2. Massively Parallel Reporter Assays

**Figure 1:**
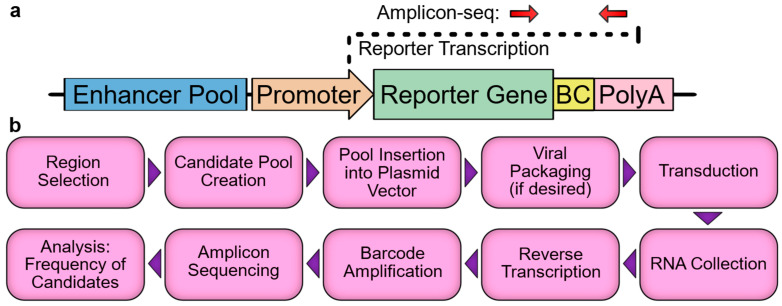
MPRA overview. (**a**) Schematic of a typical MPRA vector. Red arrows indicate PCR priming sites. BC, barcode. (**b**) Overview of MPRA workflow.

As modern genomics identifies ever increasing numbers of candidate *cis*-regulatory elements, the validation of candidates has become a major bottleneck in the field. While the activities of small numbers of candidates can be measured in vivo in mouse via the germline integration of reporter constructs, and larger numbers of candidates can be tested in vitro via transfected reporter constructs, a true high-throughput solution (the MPRA) has only recently been developed. MPRAs utilize a next-generation sequencing-based readout, which allows for many thousands of reporter constructs to be measured simultaneously in the same sample. There are several variations of the MPRA, but the key feature in each is that the regulatory element, or a barcode corresponding to the element, is embedded within an untranslated region (UTR) of a reporter gene such that the element will drive the transcription of itself or its linked barcode (Figure 1a), which subsequently will be referred to as an enhancer-identifier. Thus, when a pool of regulatory elements is assayed in an MPRA vector, the reporter gene RNA transcripts will contain enhancer-identifiers in proportion to each enhancer’s relative strength. The relative frequency of each element can be measured by the reverse transcription, amplification, and sequencing of the portion of the transcript containing the enhancer-identifiers. To account for the starting frequency of each element in the pool, the vector DNA is amplicon-sequenced, and the activity for each element is then expressed as the RNA:DNA ratio or a derivative thereof. While a number of tools and guides can be referenced during MPRA experimental design and analysis [27,28,29,30], the basic framework is fairly simple (Figure 1b) and should be well understood before undertaking an experiment. To that end, here, we provide a practical overview of the major steps and pitfalls associated with designing and executing an MPRA, with particular attention to cardiovascular and in vivo applications. We focus primarily on enhancers, as they have been the subject of the majority of the relevant literature; however, the MPRA vectors that we discuss can be easily modified for the analysis of promoters, with very few conceptual differences in the experiment.

### 2.1. Experimental Design: Assay Configuration and Context

**Figure 2:**
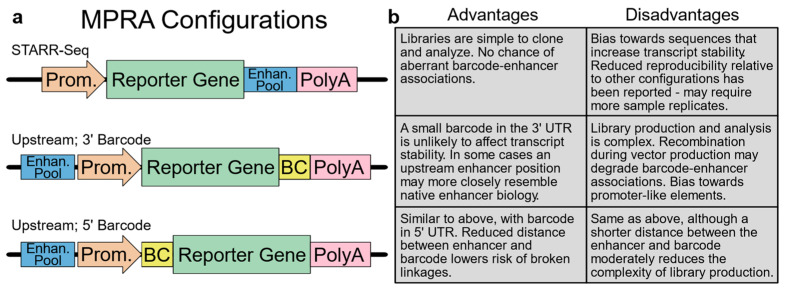
Comparison of different MPRA strategies. (**a**) Common MPRA configurations. (**b**) Strengths and weaknesses of each configuration. Prom., minimal promoter; Enhan. Pool, pool of candidate enhancers; BC, barcode.

Choosing an appropriate variant of the MPRA assay is a key step in experimental design that will impact the production and cloning of the *cis*-regulatory element library, as well as how the results are analyzed (Figure 2). Several configurations of the MPRA vector have been reported and characterized [26]. One common configuration features the insertion of an enhancer pool in the 3′ UTR of the reporter gene. In this approach, termed self-transcribing active regulatory region-sequencing (STARR-seq) [31], the enhancer sequence functions as its identifier so that enhancer identity and activity can both be determined by the sequencing of the reporter transcript. While elegant, the STARR-seq approach may be biased by enhancer sequences that affect reporter–transcript stability, and it has also been reported to suffer from elevated sample-to-sample variation [26]. Two additional common configurations feature an upstream position for candidate enhancers, which are linked with a barcode positioned in either the 5′ or 3′ reporter gene UTR [32,33,34]. Since a small barcode in the UTR is unlikely to affect transcript stability, these configurations are commonly regarded as more rigorous than STARR-seq; however, these assays may bias towards promoter-like elements [26]. Furthermore, library construction is more complex, often requiring multiple cloning steps that must maintain library diversity and the integrity of the enhancer–barcode links [35,36,37,38]. Likewise, the analysis of the data may also require significant additional expertise, depending on the strategy used for barcoding. When these three configurations were directly compared using an integrating vector, the reproducibility of the 5′ and 3′ barcoding approaches was excellent, with Pearson correlations between replicates exceeding 0.95, while the STARR-seq approach yielded moderately lower correlations exceeding 0.85 [26]. Interestingly, when this assay was repeated in a mutant non-integrating lentiviral vector, the correlations between the replicates dropped sharply for STARR-seq (to ~0.5) and moderately for the 3′ barcoding approach (to ~0.8). These results indicate that when executed with care, all three approaches are viable, but the STARR-seq approach may require additional replicates to establish comparable statistical power.

As demonstrated in the above-mentioned study, vector context is an important consideration for any MPRA system. Assays are commonly conducted within vectors that are either episomal, such as a transient plasmid transfection and an adeno-associated virus (AAV), or chromosomally integrated via lentivirus. With an integrating vector, the chromatin state likely is more representative of that of the native enhancers, although locus-dependent integration effects may add noise to the dataset. Some significant differences between integrated and episomal assays have been noted [26,39]. However, in these studies, Pearson correlations between episomal and integrated contexts typically exceeded 0.8, suggesting that episomal assays are sufficient to capture most of the valuable signals. This is encouraging since, as the field moves toward in vivo MPRAs, many cells that are poorly transduced by lentivirus, such as cardiomyocytes, are robustly transduced by AAV. In conclusion, while configuration and integration state should be chosen for maximum alignment with features of the model system and available expertise, all common MPRA configurations have been used successfully in a variety of contexts.

### 2.2. Experimental Design: Library Construction, Data Collection, and Analysis

Library construction begins with the design of the enhancer pool. Candidate regulatory elements can be selected from any number of sources, including regions of interest from ChIP-sequencing, chromatin accessibility, DNAse hypersensitivity, non-coding genomic variants from clinical sequencing data, conserved non-coding sequences, or published enhancer atlases that integrate multiple sequence features [40,41,42,43]. In addition to candidate regions, positive and negative controls should also be included. Negative controls may originate from a variety of sources, including candidate enhancers from a different cell type, random genomic sequences, or regions individually validated as being inactive. Base shuffling of candidate regions is an attractive option as it preserves nucleotide frequencies. When choosing negative controls, it is important to include a robust number of regions (typically at least several hundred) in order to later set a meaningful cut-off when categorizing candidates as “active” or “inactive”. In the past, we have identified active enhancers as those with activity greater than 95% of that of the negative controls (i.e., a 5% false discovery rate) [42,44]. Positive controls should consist of regions previously validated as having activity in sufficient numbers to instill confidence in the assay; this group typically consists of fewer regions than the negative control group. Finally, the number of replicates per sequence is an additional consideration. While many studies use only a single replicate, designing each candidate sequence to be produced in combination with multiple barcodes allows for multiple measurements per candidate within each sample, which improves the statistical power of subsequent analyses.

**Figure 3:**
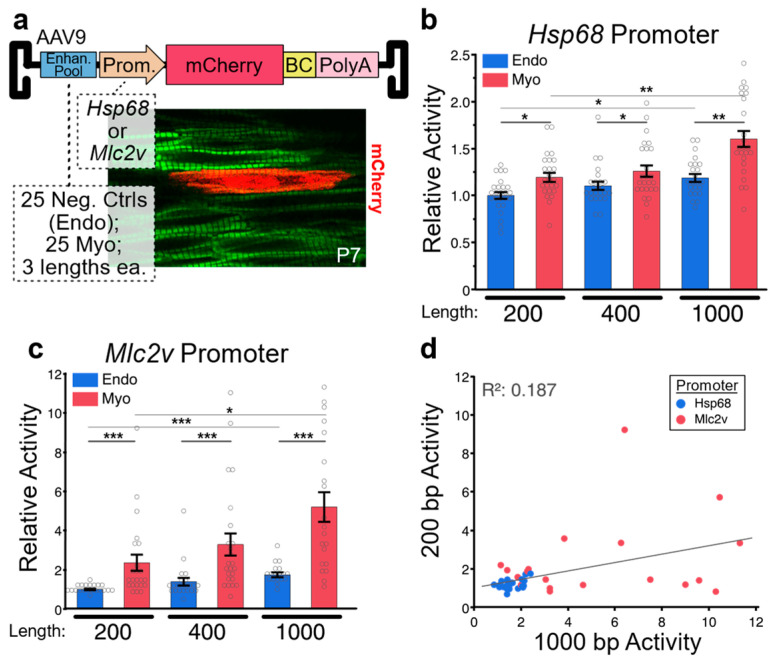
Effect of Enhancer Length on Activity (**a**) MPRA configuration. A library of 50 enhancers, each tested in three different lengths and with two different promoters (300 combinations), was packaged into AAV9 and delivered to newborn mice. Enhancers were selected from the VISTA Enhancer Browser of transgenic reporter data, and included 25 candidates active in the embryonic myocardium and 25 negative control candidates active in embryonic endothelium but not in myocardium. In the heart, AAV9 selectively transduces cardiomyocytes. Imaging of hearts from mice injected with the MPRA pool identified cardiomyocytes (green; Myh7^YFP^) with robust reporter expression (red; mScarlet) scattered throughout the myocardium. After collecting ventricles at P28, the reporter transcripts were sequenced, and the frequency of each barcode was compared to its frequency in the viral pool DNA. (**b**) When combined with an *Hsp68* promoter, average myocardial enhancer activity was higher than endothelial enhancer activity at all lengths. Within both enhancer groups, longer enhancers generally displayed higher activity than shorter enhancers. (**c**) When combined with an *Mlc2v* promoter, average myocardial enhancer activity was again greater than endothelial enhancer activity at all lengths; however, the difference between the two enhancer groups was much more pronounced. Within both groups, longer enhancers once again displayed increased activity. (**d**) Correlation between 200 bp and 1000 bp activities for myocardial enhancers. Activities (RNA:DNA ratios) were normalized to the 200bp endothelial group average. Steel–Dwass *p* < 0.05 *, *p* ≤ 0.001 **, *p* ≤ 0.0001 ***.

After regions have been selected, the size of the regions to be assayed must be chosen. Fragment size may depend in large part on the method that will be used to produce the regions. While some methods, such as error-prone PCR or region capture via array-based probes, can generate enhancer libraries with fragment lengths exceeding 1 kb [45], the upper limit of region size is commonly dictated by the constraints of pooled oligo synthesis, which currently sit at 350 bp [46]. If the chosen strategy incorporates barcodes and/or priming sites, region size is limited to ~300 bp. However, multiple groups have presented methods for the assembly of overlapping oligos to generate enhancers of increased length. A recent Nature Methods study demonstrated that two oligos can be assembled to produce 354 bp enhancers, or three oligos for 678 bp enhancers [26], while our own work has demonstrated the assembly of two oligos to produce 400 bp enhancer pools [42]. Unfortunately, few studies have systematically investigated how enhancer length affects reporter assay results, with the aforementioned Nature Methods study being the best available data. This study, which was conducted in cultured Hep2G cells, compared the activities of 651 candidate enhancers at three different lengths: 192 bp, 354 bp, and 678 bp. The authors observed a Pearson correlation between 192 bp and 678 bp enhancers of 0.53, indicating substantial differences in activity between different length enhancers. However, the direction of the difference varied from enhancer to enhancer, and significant differences in mean group activity level for different length enhancers were not observed. Surprisingly, no significance difference was reported between positive and negative controls in the 678 bp group, while significant differences were observed for the two shorter groups. To provide some clarity on the effect of enhancer length on MPRA results, we conducted a similar experiment featuring 50 enhancers that had been validated for activity in transient transgenic mouse embryos, individually synthesized in 200 bp, 400 bp, and 1000 bp lengths. Enhancers were pooled and cloned into an AAV9 reporter vector containing a minimal generic *Hsp68* promoter or a short promoter sequence from the cardiac sarcomere gene *Mlc2v* (Figure 3a). In the heart, AAV9 selectively transduces cardiomyocytes. Of the 50 enhancers tested, 25 were cardiomyocyte positive control enhancers, and 25 were negative controls that displayed endothelial/endocardial specific activity in transgenic reporter mice. For the *Hsp68* promoter-containing vector, we observed that on average, positive controls had significantly higher activity than negative controls for all lengths, with activity increasing as enhancer length increased (Figure 3b). For the *Mlc2v* promoter-containing vector, we observed similar results; however, the magnitude of the difference between positive and negative controls (i.e., the dynamic range of the assay) was considerably larger (Figure 3c). Next, we analyzed the correlation in activity between 200 bp and 1000 bp myocardial enhancers (Figure 3d). We observed a positive, albeit weak, correlation (Pearson correlation = 0.19). Enhancers with elevated activity at one length tended to also have elevated activity at the other length. However, not all enhancers followed this trend, with several enhancers showing high activity only within the 1000 bp group. Importantly, no enhancers had high activity in the 200 bp group and low activity in the 1000 bp group. Since activating motifs are much more frequent than repressive motifs, longer enhancers typically have similar or greater activity than truncations. Thus, our observations are consistent with expectations based on known enhancer biology. Interestingly, for both assays, as enhancer size increased, we observed a modest but significant increase in the activity of negative controls, in addition to the increase observed in positive controls. Thus, in both the *Hsp68* vector and the *Mlc2v* vector, the ratio between positive and negative control activities did not dramatically change as enhancer size changed, suggesting that a wide range of enhancer sizes can be effectively used in MPRA assays, with larger enhancers having higher absolute levels of activity but only a moderately improved dynamic range. Our results suggest that the choice of a promoter that has a high likelihood of robust compatibility with the candidate enhancers should be carefully considered, as should to enhancer size.

After enhancer selection and enhancer size choice, regions are typically generated by pooled oligo synthesis, PCR amplified, and cloned into the MRPA vector. As with any library amplification, it is important to use the minimum necessary number of PCR cycles to avoid mutations, maintain pool diversity, and avoid a recombination that degrades enhancer–barcode links [35]. Emulsion PCR, in which template molecules are segregated into small aqueous droplets in oil for highly parallelized amplification, is another technical approach that can minimize these issues [47]. After insertion of enhancers into the MPRA vector, sufficient plasmid must be generated for transient transfection or virus production. This typically involves the electroporation of a ligation product and the collection of a large number of bacterial colonies. In order to maintain library diversity, it is critical to collect a sufficiently large number of colonies, with greater than 100x more colonies than library sequences being ideal [48]. At this point, library plasmids should be amplicon-sequenced to verify the sufficient representation of most candidates. Candidates with poor representation in the plasmid or viral library will be excluded from subsequent analyses of RNA samples. Following the creation of the vector pool, cells are transfected or transduced. Adequate coverage of the library requires that each unique sequence be sampled many times, with 500x being a commonly referenced benchmark [49]. As an example, given that the mouse heart contains ~2 million cardiomyocytes [50], if 70% of the cardiomyocytes are transduced, and we conservatively estimate one viral particle per transduced cell, then a library of 14,000 *cis*-regulatory elements will require at least five mice for adequate coverage (500 × 14,000 = 2,000,000 × 5 × 0.7). Since samples are often relatively easy to acquire compared to the effort necessary for creation of the pooled vector, we recommend erring on the side of caution and collecting replicates sufficient for a very high coverage of the library. In our experience, too few sample replicates, both biological and technical, is a common source of noise in MPRA data.

Upon collecting total RNA, the reporter transcript is reverse transcribed, often adding a unique molecular identifier (UMI) to each molecule in the process. Next, the barcode-containing region is PCR amplified and sequenced. After the removal of PCR duplicates using UMI information [51,52], barcodes are counted and associated with their linked enhancers. After normalizing for sequencing depth, the frequency of each enhancer in the RNA-derived samples can be compared to its frequency in the starting DNA pool. This RNA:DNA ratio serves as an activity measurement that can be compared between enhancers and experimental conditions. While this analysis strategy can be refined in various ways [29,30], the basic framework is simple and accessible to most scientists familiar with transcriptomics data.

### 2.3. In Vivo Applications

A key limitation of many reporter assays is that they are often conducted in cultured cell types that have dubious relevance to in vivo biology. However, progress is rapidly being made in adapting MPRAs to in vivo use. While the first in vivo MPRA was conducted in the mouse liver via the hydrodynamic tail vein injection of plasmid [34], the delivery of MPRA pools to other tissues has been more challenging and has lagged behind. One notable system, the delivery of plasmid pools via the electroporation and culture of explanted newborn mouse retinas, has been used successfully in multiple MPRAs [53,54,55,56], shedding light on photoreceptor gene regulation. However, this in situ approach has limited applicability to tissues beyond the retina, and thus, the development of viral vectors for MPRAs is an important front in the effort to adapt MPRAs to diverse tissues. To date, much of the relevant activity has been within the neuroscience field. In 2016, an MPRA library was successfully packaged into an AAV9 variant capable of high-efficiency neural transduction, and the activities of approximately 3500 *cis*-regulatory elements were assessed within a mouse cerebral cortex [45]. This study demonstrated that reporter RNA and DNA could be recovered from transduced tissue, and it allowed for insights into the sequence features that mediate *cis*-regulatory element activity in the mouse cortex. In 2019, the work was followed up by a study that combined an MPRA with single-cell RNA-sequencing, which allowed for the resolution of enhancer activities across the different cell types that were transduced by the injection of AAV into the mouse cortex [57]. More recently, a group screened a library of candidate brain enhancers and regions associated with GWAS studies of epilepsy and schizophrenia using an AAV MPRA vector injected to the postnatal mouse forebrain. Many putative regulatory elements were validated as forebrain enhancers, including a *Cacna1c* intronic region previously associated with neuropsychiatric disorders [58].

Despite the existence of AAV vectors with strong cardiomyocyte tropism, the cardiac adoption of MPRAs has lagged. To date, only two cardiac studies have been published, both from the laboratory of Dr. William Pu. The first, our work with genomic regions bound by multiple core cardiac transcription factors [42], featured a library of 2700 regions, each 400 bp in length, generated by annealing overlapping oligos. Regions were cloned into the 3′ UTR of a scAAV *Mlc2v* promoter-containing reporter vector (Addgene #182649), which was packaged into the AAV9 capsid and delivered to newborn mice. A week after injection, the hearts were collected and enhancer activities were measured. On average, enhancers bound by multiple core cardiac transcription factors displayed robust activity, while negative control regions corresponding to putative mouse embryonic stem cell enhancers did not, thus confirming the importance of core cardiac transcription factor binding for transactivation in cardiomyocytes. The second cardiac study expanded on these results by using a similar ChIP-seq-based approach to identify candidate enhancers, including a subset with atrial- or ventricular-specific occupancy [44]. Chamber-specific and non-specific candidates were then assayed by MPRA in the atria and ventricles. This strategy featured 2943 candidate enhancers and 954 negative control regions, each 400 bp in length, constructed via the pooled synthesis of self-priming oligo pairs. Candidates were cloned into a STARR-seq style AAV-MPRA vector and assayed. Of the candidates, 1092 had activity in either the ventricle or atria, with the activity of 229 enhancers having significant chamber specificity. From the active enhancers, subsets of chamber specific and non-specific enhancers were selected for dense mutagenesis. This was achieved by the pooled synthesis of a series of shorter sequences “tiled” at 5 bp overlapping intervals across the larger enhancer. For each tile, a wildtype version and a mutant version were synthesized, with the mutant having a deletion of the central 5 bp. Thus, by assaying these wildtype and mutant tiles in both the atria and the ventricle, specific motifs conferring activity and chamber specificity were discovered. One such motif, ERRα/γ, was shown to be necessary for ventricle specific activity in a subset of enhancers, with subsequent studies showing that ERRα/γ double knockout in cardiomyocytes results in the loss of ventricular identity. By rapidly screening large numbers of candidates and finely dissecting those with chamber-specific activity, this study effectively demonstrated the utility and versatility of in vivo MPRAs.

## 3. Discussion and Perspectives on Future Directions

While the studies mentioned above demonstrate the feasibility and value of using AAV to deliver MPRA libraries, this approach remains in its infancy. In the near future, we expect a surge of studies spanning diverse organ systems, with the heart being well represented. We anticipate that the future of cardiac genomics will include a much more comprehensive MPRA-based characterization of enhancers, including measurements of activity across development and during disease. As enhancers of interest are identified, high-resolution dissection will identify the molecular mechanisms underlying their activity profiles. Traditionally, enhancer dissection has been achieved using reporter assays with truncated versions of the enhancer, while the MPRA era has made saturating mutagenesis possible. In this approach, all parts of the enhancer are independently mutagenized and tested, allowing for a more comprehensive analysis [32,59,60,61]. Mutagenesis can be achieved through error-prone PCR, or as mentioned above, synthesis of a series of enhancers in which each features a different mutation or group of mutations. In either case, mutant enhancers that show a loss of activity relative to the wildtype element can be analyzed to identify the crucial motifs, giving important clues as to which protein regulators are responsible for transactivation. Alternatively, the use of bioinformatics to identify candidate motifs for targeted mutation allows for the collection of mechanistic data while greatly reducing the necessary size of the regulatory element library. This targeted approach has been used effectively to analyze the impact of specific motif families on enhancer activity [62], and we expect this to be a fruitful strategy for characterizing the roles played by various cardiac transcription factors and their corresponding motifs.

While MPRAs are a powerful tool, they have several limitations. First, it can be challenging to directly link MPRA data to gene expression. Enhancers can be located long distances away from the genes they regulate, and thus, integrated analysis with high-resolution chromatin interaction maps may be required to make these regulatory connections. Furthermore, an enhancer that is sufficient to activate the expression of a reporter gene may not be necessary for gene expression in the native genomic context. Indeed, enhancer redundancy is a commonly observed adaptation that ensures robust and resilient gene expression [63,64,65]. Similarly, specific motifs that are necessary for enhancer activity may not be necessary for gene expression. While these issues can be addressed using the traditional approach of generating a mouse line with a targeted mutation, the growing popularity of CRISPR-mediated somatic mutagenesis in the heart potentially offers an expedient alternative [66,67,68]. In such a system, Cas9-expressing mice are transduced with a gRNA targeting a motif of interest. The resulting targeted double strand breaks are repaired by error-prone non-homologous end joining, which typically produces a small mutation sufficient to disrupt a regulatory motif. After confirmation of successful mutagenesis, the expression of the associated gene can be assessed. While this approach may not be appropriate for all motifs, such as those that lack a suitable gRNA PAM sequence, this will allow for the targeting of most motifs in a scalable manner.

A second limitation of MPRAs is the inaccessibility of many tissues and developmental timepoints. While postnatal cardiomyocytes are easily transduced by AAV9, non-myocyte cardiac populations are challenging. However, a number of groups are pursuing large-scale AAV capsid engineering [69,70,71], and many variants with increased tropism for previously poorly transduced cell types have already been developed [72,73,74]. In addition to the challenges of transducing diverse cell types, many of the most dynamic gene regulatory events within the heart take place during embryonic development. While AAV and other popular viral vectors administered during pregnancy typically do not cross the placenta or fetal membranes [75], the direct fetal injection of AAV has shown variable levels of success in transducing a variety of embryonic tissues [76,77,78,79,80], including high-efficiency transduction of the myocardium by AAV9 during late gestation [81]. While the early stages of heart development may be inaccessible for the foreseeable future, we expect that the improvement in viral vectors and delivery protocols will continue to accelerate, eventually allowing access to most cardiac cell populations at a range of timepoints, including mid to late gestation.

A third limitation of MPRAs related to AAV vectors is the uncertain chromatin state of the reporter vector. After cellular import, AAV genomes are converted from single- to double-stranded DNA. AAV genomes then persist as circularized monomeric or concatemeric extrachromosomal episomes, which acquire chromatin properties that include a typical nucleosomal pattern [82,83]. Nevertheless, it is not clear if vector-derived chromatin receives the full set of typical modifications, raising the possibility that episomal vectors may not be able to fully recapitulate the activity profiles of some enhancers. In comparison, lentivirus vectors integrate into the host genome and can be marked by the full set of epigenetic modifiers; however, integration position effects will introduce variability to the assay results. One potential improvement on current approaches is to employ site-specific insertion of the reporter vector into the host genome. While this strategy has been successfully executed for individual enhancers in genetically modified mice [84], an MPRA has not been possible. However, developments in genome editing will likely change this in the near future. Recently, we showed that AAV vectors carrying donor templates can facilitate the CRISPR-mediated precise integration of transgenes within the genomes of postnatal mouse cardiomyocytes via homology-directed repair [81]. A similar approach could be used to precisely insert a library of cis-regulatory elements at a target locus in vivo. We eagerly look forward to such developments, which may improve the accuracy, reproducibility, and dynamic range of MPRAs.

In summary, MPRAs are a powerful tool for the high-throughput assessment of cis-regulatory element activity, and the adaptation of this tool for use in vivo is particularly exciting, given the time and budgetary constraints of traditional methods of enhancer evaluation. MPRAs have a range of applications, including identification of novel cis-regulatory elements, dissection of known elements, characterization of element activity across development or during disease, and characterization of disease-associated variants. MPRAs can be deployed in a variety of configurations with varying levels of complexity, and we anticipate that MPRAs will soon be used in combination with other functional genomic techniques to systematically characterize the transcriptional networks that govern cardiovascular gene expression.

## Data Availability

The data presented in this study are openly available in the Zenodo repository at 10.5281/zenodo.7779156.
